# Reproductive Biology in the Possible Last Healthy Population of *Parodia rechensis* (Cactaceae): Perspectives to Avoid Its Extinction

**DOI:** 10.3390/plants13202890

**Published:** 2024-10-15

**Authors:** Rafael Becker, Rosana Farias-Singer, Diego E. Gurvich, Renan Pittella, Fernando H. Calderon-Quispe, Júlia de Moraes Brandalise, Rodrigo Bustos Singer

**Affiliations:** 1 Graduate Program in Botany (PPGBOT-UFRGS), Universidade Federal do Rio Grande do Sul, Porto Alegre 90035-003, RS, Braziljuliabrandalise05@gmail.com (J.d.M.B.); rbsinger1@yahoo.com (R.B.S.); 2 Porto Alegre Botanical Garden, Secretaria do Meio Ambiente e Infraestrutura do Estado do Rio Grande do Sul, Porto Alegre 90119-900, RS, Brazil; 3 Cátedra de Morfología Vegetal & IMBIV (FCEFyN), Universidad Nacional de Córdoba, Córdoba 5000, Argentina

**Keywords:** breeding system, cactus, conservation, mellitophyly, pollination

## Abstract

All 32 Brazilian species of *Parodia* Speg (Cactaceae) occurring in Rio Grande do Sul State are considered threatened, according to the IUCN criteria. Until 2021, *Parodia rechensis* (CR) was known by only two small populations. However, a new population with over 400 individuals was discovered in 2021, prompting the study of its reproductive biology as a way to promote its conservation. Anthesis, breeding system, and natural pollination were studied in the field. The breeding system was studied by applying controlled pollination treatments to plants excluded from pollinators (bagged). Germination features were studied at the Seed Bank of the Porto Alegre Botanical Garden under controlled temperatures (20, 25, and 30 °C). The anthesis is diurnal and lasts for up to four days. The flowers offer pollen as the sole resource to the pollinators. The study species is unable to set fruit and seed without the agency of pollinators and has self-incompatible (unable to set fruit and seeds when pollinated with pollen of the same individual) characteristics that can considerably restrict its reproduction. Native bees of Halictidae and Apidae (Hymenoptera) are the main pollinators, with a smaller contribution of Melyridae (Coleoptera) and Syrphidae (Diptera). Natural fruit set is moderate (≤64%, per individual), but the species presents vegetative growth, producing several branches from the mother plant. Seeds showed the optimum germination rate at 20 °C and an inhibition of 75% in germinability at 30 °C. Our findings suggest the need to manage the species’ habitat to guarantee the permanency of the plants and healthy populations of pollinators as well. Our findings raise concerns about the germination and establishment of new individuals in the context of rising temperatures caused by climate change. Suggestions for the possible management of the extant populations are made.

## 1. Introduction

As currently accepted, *Parodia* Speg. (Cactaceae) embraces 62 species [1]. This genus has one of its centers of diversity in southern Brazil, with particular prominence in the State of Rio Grande do Sul, where 32 species occur [2]. A characteristic that makes this genus a remarkable element within the biodiversity of Rio Grande do Sul is its endemicity: fourteen species of *Parodia* occur exclusively in the state [2], some of which have very restricted distributions. This pattern of restricted distribution, together with man-promoted threats (e.g., habitat loss and illegal collection), leads to a situation where all of these endemic species are classified at some level of threat of extinction according to IUCN criteria [2]. *Parodia rechensis* (Buining) F. H. Brandt is currently categorized as Critically Endangered (CR) by the IUCN [3] and is, arguably, one of the most threatened Brazilian cacti. The plant has an extremely restricted distribution in the Municipality of Caxias do Sul, being one of two cactus species endemic to Rio Grande do Sul that exclusively occurs within the Atlantic Forest domain [2,4]. Historically, only two populations were recognized in the Municipality of Caxias do Sul, distant about 8 km from each other [1]. Both populations were probably larger and connected in the past, since the area was heavily affected by the construction of a dam. Due to being widely disseminated in online databases, both populations have suffered from illegal and unrestricted collection of their individuals, leading to a 75% reduction between 2004 and 2011 [5]. Currently, the two populations combined have six individuals (Becker, pers. obs.). However, in October 2021, an unexpected finding brought some hope: researchers based at the Porto Alegre Botanical Garden located a new population approximately 15 km away from the already known populations. This new population holds about 450 individuals, making it the largest remaining population. However, this recently found population needs to be urgently studied to properly address the biological features that could help to improve its situation, as, even with this new finding, the species still remains restricted to the same region and the threats may persist.

**Figure 1:**
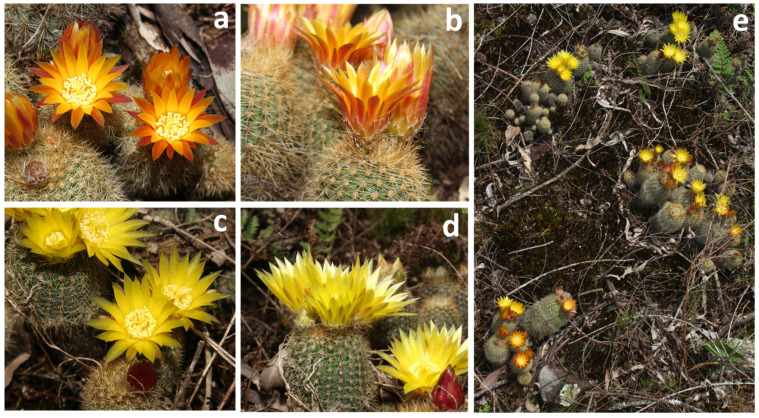
General appearance of *Parodia rechensis* in its habitat. (**a**,**b**) Flowers with orange perianth elements. (**c**,**d**) Flowers with yellow perianth elements. (**e**) Plants of both phenotypes in the environment.

*Parodia rechensis* was originally described in 1968 under the name *Notocactus rechensis* Buining. The plant has a cespitose habit; the stems are globose to short-cylindrical, with slightly evident ribs and yellow to white spines (one darker central spine and up to 17 radials). The flowers are showy, up to 25 mm in diameter. The tepal’s coloration varies from greenish to yellow, and the external elements can be yellow or orange (Figure 1). The stigma is creamy-white colored, and the stamens are yellow [2]. The plant exhibits pronounced growth through budding, producing a large density of branches. *Parodia rechensis* shares a set of vegetative features (e.g., shape of ribs and spines) with *Parodia alacriportana* Backeb. and Voll, *Parodia haselbergii* (Haage ex Rumpler) F. H. Brandt, and *Parodia graessneri* (K. Schum) F. H. Brandt. However, the floral morphology in *P. rechensis* is different, with a wider calyx opening and mostly yellowish tepals, which hypothetically enable pollination by different animal groups, unlike *P. haselbergii* and *P. graessneri*, where the flowers have a narrow tube and tepals with different colors and specialized pollination strategies [1,2].

**Figure 2:**
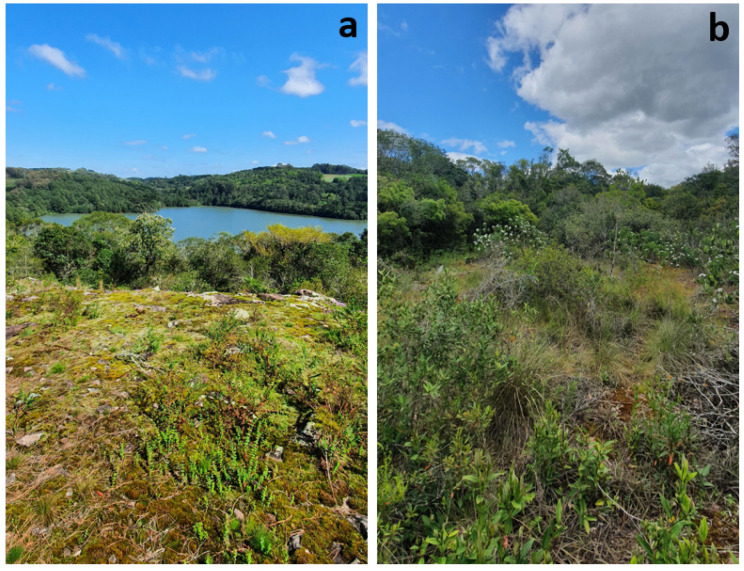
*Parodia rechensis* habitat. (**a**) Area of the type population discovered in 1968. (**b**) New population discovered in 2021.

Like most cacti in southern Brazil, *P. rechensis* is found in rocky outcrops, where it forms clusters that make it difficult to determine whether the origin of the individuals was through budding or seeds. The plants occur in rocky outcrops areas exposed to the sun, but it was observed that shrubs are growing over the outcrops, shading the plants (Figure 2). This population is situated within the Mixed Ombrophilous Forest, part of the Atlantic Forest domain, characterized by the abundant presence of *Araucaria angustifolia* (Bertol.) Kuntze and has a high average annual precipitation of 1802 mm, ranging from 111.3 mm to 189.4 mm between the least and most rainy months [6]. Additionally, the southern region of Brazil has been experiencing an increase in average precipitation, raising the volume of rainfall and the frequency of extreme events [7,8]. This gradual increase in precipitation, caused by climate change, may bring changes in the floristic composition, converting drier environments, such as rocky outcrops or grasslands, into shrub or forest formations [9]. In this context, and as a way to preserve the extant populations of *P. rechensis*, it is important to evaluate the possible effect of vegetation changes on species performance in order to evaluate possible management practices (e.g., partial vegetation management or vegetation removal). It is observed that parts of the rocky outcrop where the new population of *P. rechensis* is located are covered by native shrub vegetation and forestry (*Eucalyptus* sp. and *Pinus* sp.) (Figure 2b). The conversion to a more shaded environment can significantly impact species adapted to sunnier or open environments and alternating herbaceous diversity [10,11,12].

The present study aimed to uncover the processes of sexual reproduction and the pollination biology of this species to better understand the reproductive requirements of this species. Since it is a critically endangered species with an extremely restricted distribution and scarcely known individuals in the wild, reproductive studies are necessary. With the discovery of the new population of *P. rechensis*, it is now possible to understand the reproductive dynamics of the species. The main questions of this study are: (1) Does the species have a self-compatible breeding system (as in other *Parodia* spp. studied in precedence) and pollinator-dependent? (2) Which pollinators are involved in pollen transport? (3) What is the germination profile, and what is the ideal germination temperature of the species? (4) Do individuals in shaded locations have different vegetative growth than individuals exposed to the sunlight? By resolving these questions, it is possible to propose more accurate conservation strategies for this species, either by working directly with the new population or by cultivating ex situ to reintroduce individuals into other populations that have already been impacted. Our hypotheses are that (1) *P. rechensis* would be self-compatible and pollinator-dependent; (2) pollen-seekers bees are involved in the pollen transport; (3) higher temperatures would affect germination significantly; and (4) sunlight would favor vegetative growth and branch production.

## 2. Results

### 2.1. Anthesis and Pollination Observations

**Figure 3:**
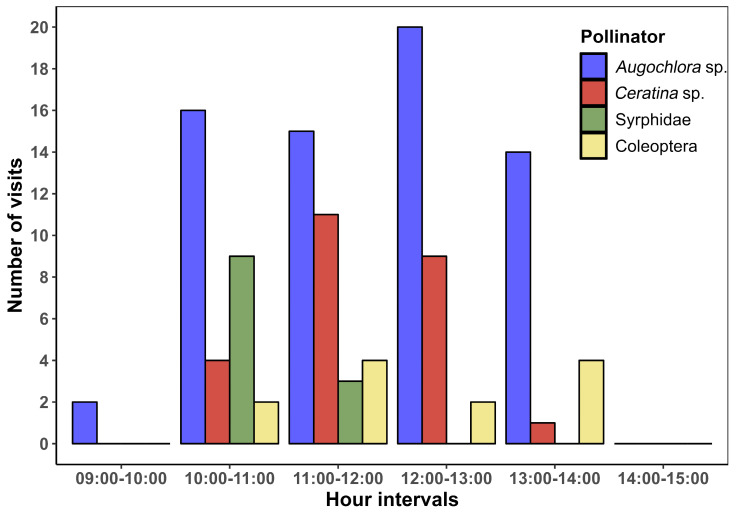
Average frequency of pollinator interaction with *Parodia rechensis* flowers at 60-min intervals.

**Figure 4:**
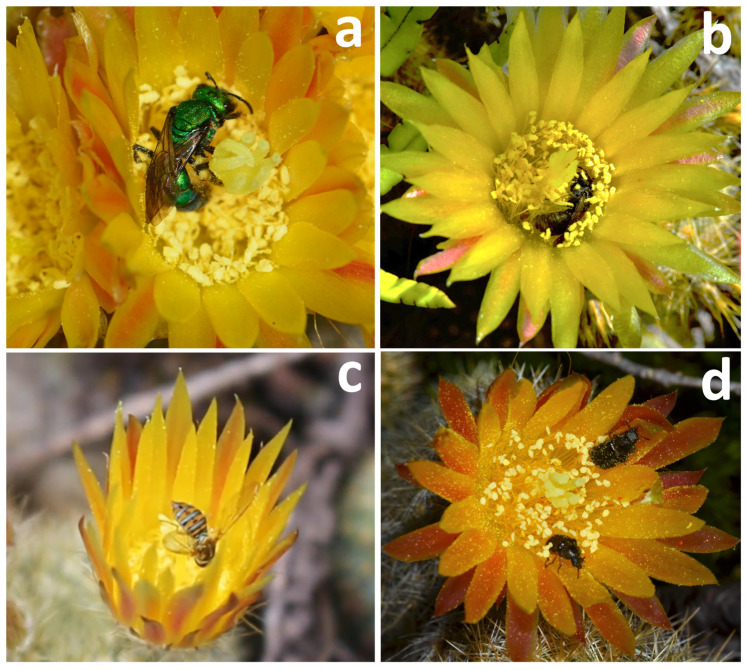
Insect pollinators in *Parodia rechensis*. (**a**) *Augochlora* sp. (Halictidae); (**b**) *Ceratina* sp. (Apidae); (**c**) Syrphidae; and (**d**) Melyridae.

The anthesis of *P. rechensis* starts around 9:00 AM and extends until 3:00 PM. Insect visits are concentrated between 10:00 AM and 2:00 PM, being rare in the first and last hour of anthesis (Figure 3). The resource offered by the flowers of *Parodia rechensis* is pollen, produced in large quantities. No nectar production was observed. Four distinct species of insects were observed interacting with both sexual whorls: *Augochlora* sp. (Hymenoptera, Halictidae), *Ceratina* sp. (Hymenoptera, Apidae), Sp. 1 (Diptera, Syrphidae), and Sp. 2 (Coleoptera, Melyridae) (Figure 4). A fifth species of *Camptodes* (Coleoptera, Nitidulidae) was sporadic and frequently hid inside the flowers in our presence. Thus, among the Coleoptera, only Melyridae behavior could be recorded and quantified in detail. The four pollinator species observed interacting with the flowers exhibited foraging and/or pollen collection behavior. The number of interactions varied among the pollinators (Table 1). Bees (*Augochlora* sp. and *Ceratina* sp.) had more interactions with the flowers, preferably using the stigma as a landing platform to further access the pollen. In all cases, bees were recorded actively gathering pollen. Both kinds of bees touched both sexual whorls numerous times during each floral visit. The observed Melyridae spent much more time per flower, foraging on pollen, averaging 371.88 s. In these interactions, the beetles rarely touched the stigma, with this behavior being observed only twice. Syrphidae flies were common only in the morning period and spent little time at the flowers (5.11 s on average), using the stigma as a landing platform. Unlike the beetles, the flies were rarely observed interacting with the stamens (two interactions observed, with the flies licking pollen from the stamens). Full videos of the interactions can be accessed in the Supplementary Materials.

### 2.2. Breeding Systems

The observed flower lifespan was four days, with no variation. The hydrogen peroxide test showed a pronounced reaction during the first three days and a decay at the fourth, indicating the stigmatic receptivity during almost the whole flowering period. The reproductive system tests demonstrated successful fruiting only in the two treatments: manual cross-pollination and natural pollination. On the other hand, the treatments of manual self-pollination and spontaneous self-pollination resulted in no fruit formation, indicating that the study species exhibits self-incompatibility and is pollinator-dependent (Table 2). Both the success of fruiting and seed production did not show a significant difference in the ANOVA test (*p* = 0.0658 and *p* = 0.0932, respectively) between the manual and natural cross-pollination treatments, highlighting that the behavior of the pollinators is equivalent to the fruiting capacity of the species. Fruiting success in 2023 was moderate. A total of 350 flowers were produced by the twelve monitored individuals, and 143 of them (40.85%) turned into fruit. Individually, fruiting success ranged from 3.70% to 63.33%.

### 2.3. Germination Tests

The germination tests showed that the three indexes showed a significant statistical difference in the analysis of variance (germinability: *p* = 0.0021; average germination time: *p* = 0.0056; synchronization index: *p* = 0.01204) (Table 3). Germinability decreases significantly at 30 °C compared to the 20 °C group. Nonetheless, seeds take longer average time germination at 20 °C compared to other treatments. The synchronization index was higher at 20 °C than at 25 °C. Based on the indexes, the species has the optimal temperature at 20 °C and suffers significant germination inhibition at 30 °C.

### 2.4. Vegetative Growth

**Figure 5:**
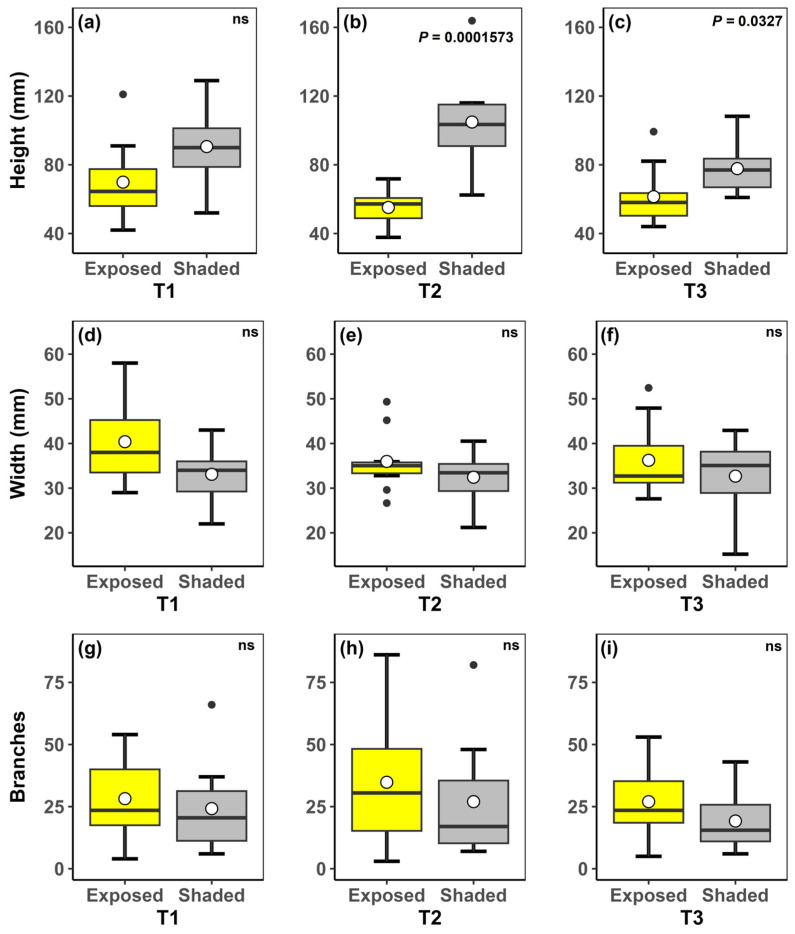
Boxplot graphics showing the variation in height (**a**–**c**), width (**d**–**f**), and number of branches (**g**–**i**) of *Parodia rechensis* during 12 months. In yellow is the group exposed to the sun, and in gray is the shaded group. T1: initial measurement (October/2022); T2: period between October/2022 and April/2023; T3: period between April/2023 and October/2023. Significant *p*-values are indicated in their respective boxplots. (ns): non-significant *p*-value at 5%.

Vegetative growth measurements over the twelve months showed few distinct variations between the T1 (initial measurement), T2 (October 2022–April 2023) and T3 (April 2023–October 2023) periods. The diameter of the plants and the number of branches in both treatments (shade and exposed) did not show a significant difference when comparing the treatments between the respective periods or the periods within the same treatment. However, the height of the plants varied. When comparing treatments in each measurement period, the shaded group showed statistically significant growth in height compared to the group exposed to the sun during periods T2 and T3 (*p* = 0.0001573 and *p* = 0.0327, respectively) (Figure 5).

## 3. Discussion

*Parodia rechensis* is a critically endangered (CR) species according to the IUCN criteria [2,3]. The only two populations known until recently had a population reduction of 75% between 2004 and 2011, with only 52 individuals remaining [1]. Today, these two populations together contain only six individuals, representing a new reduction of at least 89% between the last data (2011) and the present study (2024). The discovery of the new population, containing around 450 individuals, brings some hope for the conservation of the species, enabling for the first time the study of its reproductive biology and pollination interactions.

The pollinator species observed confirm what was expected considering the floral morphology of the species. *Parodia rechensis* presents a diurnal anthesis and is pollinated by a variety of pollen-gathering and pollen-eating insects, the main pollinators being native bees from the Halictidae (*Augochlora* sp.) and Apidae (*Ceratina* sp.). These solitary bee groups have been observed to present very specific relationships with *Parodia graessneri* [13] and *Parodia neohorstii* [14], respectively. In *P. rechensis*, solitary bees represented 79.3% of the floral visits observed, demonstrating, therefore, the important relationship of these two families of bees with the genus *Parodia*. Other pollinators observed, such as a species of Syrphidae (Diptera) and Melyridae (Coleoptera), demonstrated not very efficient cross-pollination behaviors. Syrphidae flies generally used the stigma as a landing platform, but the insects often left the flower without even touching the anthers. Beetles spent long periods within the same flower and rarely changed plants, thus probably mostly promoting self-pollination. Owing to our observations, bees are most likely to promote cross-pollination (the only experiment treatment yielding fruit). Whereas Syrphid flies and beetles were observed visiting different plants, their passive behavior at the flowers suggests that *P. rechensis* mostly relies on bees for cross-pollination and, consequently, to produce fruit and viable seed. The only resource offered to bees is pollen, with nectar production not being observed. Other *Parodia* species are nectariferous; however, in *P. haselbergii* and *P. graessneri*, very small volumes of nectar were observed [13]. It is possible that pollen be the main resource offered to the pollinators within the whole genus *Parodia*, a factor that may explain the greater number of pollinator interactions involving pollen-seeking insects already recorded [13,14,15].

The reproductive system experiments on *P. rechensis* showed that the species is self-incompatible and pollinator-dependent since manual and spontaneous self-pollination treatments did not generate fruits. This result is a novelty within the genus *Parodia*, since *P. haselbergii*, *P. graessneri* [13], *P. neohorstii* [14], *P. carambeiensis* [16], and *Parodia mammulosa* subsp. *submammulosus* (Lem.) Hofacker (Gurvich D.E.:, pers. obs) are self-compatible species, even though *P. neohorstii* and *P. carambeiensis* showed a significant decrease in manual self-pollinated fruiting success [14,16]. Fruiting success in manual and natural cross-pollination tests was considered moderate (42 and 53%, respectively), compared to fruiting success rates of other *Parodia* species, which had values between 88 and 100% [13,14,16]. In addition, natural fruiting success in 2023, as a whole, was also moderate (40.85%). The fruiting success values did not test for a significant difference between manual and natural cross-pollination (*p* = 0.0834), which shows that the pollinators’ behavior is effective despite the fruiting limitation of *P. rechensis*. These reproductive system tests highlight a problem in the sexual reproduction of *P. rechensis* since it is a self-incompatible plant and has low fruiting success through cross-pollination. The isolation of such small populations generates high inbreeding that may be causing a cost to fruit production [17,18,19]. In other words, all individuals in the *P. rechensis* population may be highly related. Thus, even cross-pollination may promote a moderate fruiting success and seed yield as a consequence of genetic relatedness. Furthermore, self-incompatibility can also make the formation of interspecific hybrids unfeasible, since self-incompatibility can reduce the probability of hybridization because it has been linked with interspecific pollen rejection [20,21]. Such hybrids could be another alternative for increasing genetic variability and could generate new adaptations, such as tolerance to climate change [22].

**Figure 6:**
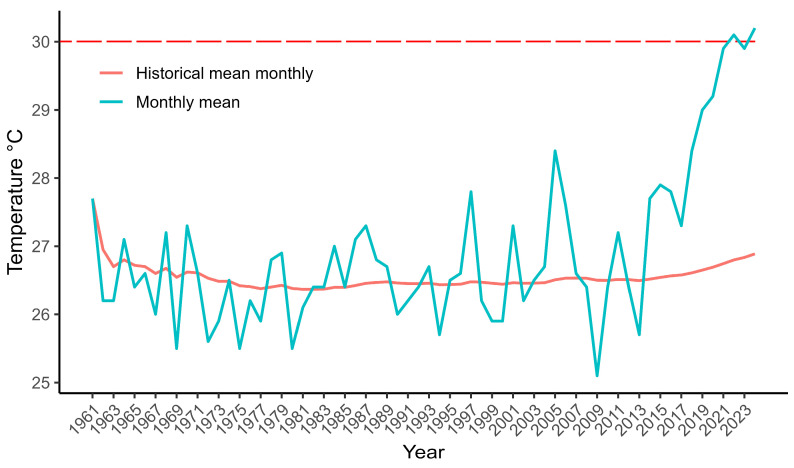
Temperature in Caxias do Sul, Brazil, between 1961 and 2024. Mean maximum temperature for October–January (blue); historical mean maximum temperature (orange) (INMET, 2024).

The germination test showed that the optimum temperature for *P. rechensis* is at 20 °C, while at 30 °C the species suffers a reduction of 58% compared to the 20 °C group. These results support the findings for *P. haselbergii* and *P. graessneri* [13], both sympatric species of the same Ombrofilous Mixed Forest. Most Cactaceae seeds show optimum temperature between 20 and 25 °C, while temperatures around 30 °C promote germination inhibition [23,24]. The new seeds of *P. rechensis* are available in nature between October and January, during the transition of spring to summer. Thus far, information regarding the viability of the seeds under natural conditions and if they can remain viable at the soil’s seed bank is lacking. Considering the period of 1961–2024, Caxias do Sul has presented an increase in mean maximum temperature for months between October and January mainly in the last decade (Figure 6). The mean maximum temperature for the period had never reached the 30 °C mark until it was surpassed in 2022 and 2024 [6]. This increase in maximum temperature during the last months of the year, caused by climate changes, may probably affect the reproductive success of the species.

In contrast to its sexual reproduction with moderate fruiting success, the species has a very evidently prolific vegetative reproduction. The plant constantly produces shoots, forming groups that can have more than 100 branches. Other species of *Parodia* are capable of branching [2]; however, it is not common, and they rarely form numerous groups like those that occur in *P. rechensis*. This factor can be considered a good starting point for adopting measures to reintroduce the species into populations where the plant is practically extinct, even if one has to consider the lack of genetic variability that a population of branches can entail.

Regarding vegetative growth, we found that shaded plants did not differ in the width and the production of new branches but grew taller than unshaded plants. The growth in height seems a plastic response of plants growing in shaded environments: plants grow taller to compensate for the shaded conditions. *Parodia rechensis* presents small and short branches, so the main indicator of growth is the production of new branches. We found no differences in the production of new branches, so the encroachment process would not have effects on plant growth. The shaded group had more significant height growth than the group exposed to the sun during the summer and winter growth intervals. This can be explained by the fact that shaded environments, where the most abundant wavelength is extreme red, cause plants to increase in height as a response to maximize light capture, since this shaded condition disfavors photosynthetic activity [25]. On the other hand, the group exposed to the sun may have suffered photoinhibition of photosynthesis caused by the stress of excessive light, exceeding the photosynthetic capacity of the species [26]. Nevertheless, comparing the growth intervals within the treatments, a reduction in the height of individuals in the shaded group was observed after the winter period (between April 2023 and October 2023). During this period, solar incidence and temperature decreased, disabling the shaded group from reaching photosynthetic optimum, while the group exposed to full sun did not exceed photosynthetic capacity, avoiding photoinhibition and experiencing positive growth. This negative effect of shading on the height of individuals has already been observed in *Melocactus bahiensis* (Britton and Rose) Luetzelb., where a loss in the length and dry mass of the root system was also observed [27]. Other negative effects may be associated with shading, such as low seed production, since a positive correlation has been observed between the number of seeds and the diameter of individuals of *Wigginsia sessiliflora* (Hook.) D.M. Porter (currently synonymous with *Parodia erinacea*). According to the authors, larger individuals play an important role in the conservation of populations, with emphasis on intermediate individuals [28]. Nonetheless, for *P. rechensis*, our data showed non-significant growth in width and branching comparing both groups. This is an indication that there is no difference in exposed and shaded environments in the growth of mature individuals. However, although we do not find differences in the vegetative growth between two situations, more long-term monitoring would be necessary, in addition to some reproductive measurements, to understand encroaching effects.

## 4. Materials and Methods

### 4.1. Study Area

**Figure 7:**
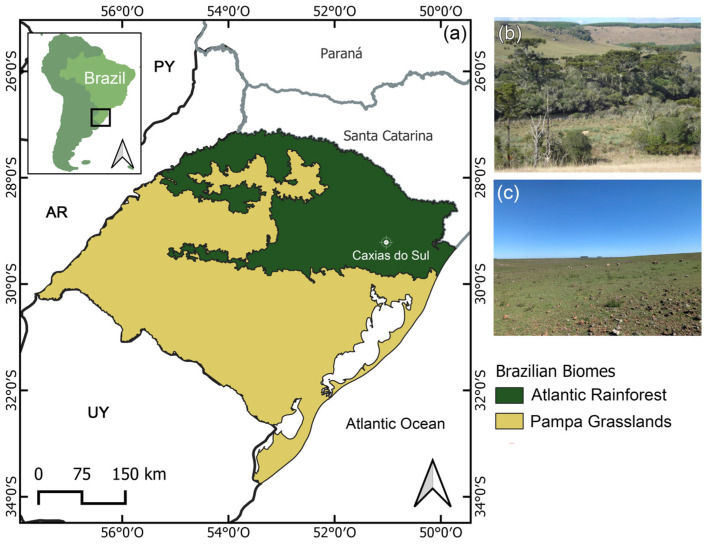
*Parodia rechensis* location. (**a**) Rio Grande do Sul state map with phytogeographic domain borders, following IBGE (Instituto Brasileiro de Geografia e Estatística). (**b**) general aspect of Mixed Ombrofilous Forest, an Atlantic Rainforest biome; (**c**) general aspect of Pampa grasslands.

This study was conducted on a recently discovered new natural population of *Parodia rechensis*, located in Caxias do Sul, Rio Grande do Sul state (29°10′04″ S, 51°10′44″ W), southern Brazil (Figure 7). For conservation purposes, the exact location of the population will be omitted, and we only indicate the coordinates of the Municipality of Caxias do Sul [29,30]. The population is situated in the Mixed Ombrophilous Forest, within the Atlantic Forest domain, with an average annual precipitation of 1800 mm and annual average temperatures ranging from 13.1 °C to 22 °C. The elevation of the municipality is around 720 to 900 masl [6]. The species has a restricted distribution, uniquely being present in the rural part of the municipality. The plants are found in rocky outcrops fully exposed to sunlight, although parts of the studied population occur within shaded shrub areas. The population has approximately 450 individuals (groups apparently arising from vegetative reproduction), currently making it the third known population and the largest in terms of the number of individuals.

### 4.2. Pollinator Observations

Anthesis and pollination observations were carried out in the field during the flowering period, between September and November, in 2022 and 2023. A total of 40 observation hours were spent on pollinators and the pollination process. The observations were made during the anthesis, approximately between 9:00 AM and 3:00 PM, during seven non-consecutive days (see Section 2). We measured nectar volume from five flowers of distinct individuals using a micropipette [31]. Observations were made on five flowers randomly selected each observation day. All insect visits were recorded on video to measure the duration of interactions with precision and determine which insects were effective pollinators or floral visitors. Only animals that touched the stigma and anthers of different flowers, transferring pollen, were considered effective pollinators. Pollinators were collected for further identification and deposited at the Museu de Ciências Natural (MCN), Porto Alegre, Brazil.

### 4.3. Anthesis, Breeding System, and Reproductive Success

In order to ascertain floral lifespan, nine flowers from specimens cultivated at Porto Alegre Botanical Garden were excluded from pollinators (bagged with tule bags) and monitored. We considered anthesis as the time interval in which the flower maintains at least one of the sexual whorls active (pollen release or stigmatic receptivity). These same flowers were also used to test stigmatic receptivity by dropping a few droplets of hydrogen peroxide onto the stigmatic surface [32]. The reproductive system experiments were conducted in situ, using voile bags to isolate the flowers from pollinators [33]. The flowers were bagged before the onset of anthesis to prevent pollen contamination from floral visitors. The flowers were tested under five treatments at the first day of anthesis: (1) manual cross-pollination (the flowers were manually pollinated with pollen from another individual located at least 20 m away); (2) manual self-pollination (the flowers were manually pollinated with their own pollen); (3) natural pollination (the flowers were only marked with a tag and left exposed to natural pollinators); and (4) spontaneous self-pollination (the flowers were only bagged without any other manipulation). At least 30 flowers per treatment, distributed among 15 individuals. About two months after the tests, the fruiting success of the treatments and the number of seeds produced per fruit were recorded. As a complementary measurement of reproductive success, in September 2023, twelve whole plants exposed to natural pollination were monitored from bud production until fruiting (in December 2023). Fruiting (as percentages) in these plants was calculated in two ways: (1) as the total number of flowers divided the total number of fruits produced, and (2) flowers produced per individual divided per fruits produced by the same individual.

### 4.4. Germination Test

Seeds produced in breeding system experiments were submitted to germination tests in a germinator under controlled temperatures. The ripe fruits of breeding system treatments were collected and dissected in the laboratory. For disinfecting the seeds, we washed it with a solution of NaClO 0.5% for three minutes and an additional two minutes in current water [34]. We repeated this washing process five times. After that, we prepared plastic boxes (Gerbox) with blotting paper moistened with 10 mL of deionized water, and each box received 25 seeds with four replicates, totaling 100 seeds for each treatment. We performed three constant temperature treatments: 20 °C, 25 °C, and 30 °C in a germinator (Tecnal TE-4020 LED). All treatments were made under a 12-h photoperiod. Germinated seeds were counted daily using a stereomicroscope. Seeds that emitted a visible hypocotyl–root axis were considered germinated. After 30 days, we ended the counting due to the stabilization of the germination curve, and we calculated the indexes of germinability (%), average time of germination [35], and synchronization index [36]. We tested the normality of data using the Shapiro–Wilk test. After that, the indexes were compared between temperature treatments by ANOVA, followed by the Tukey test at 5%. We carried out the statistical analyses using the software RStudio 2023.03.1 [37].

### 4.5. Vegetative Growth

The vegetative growth was monitored for twelve months to understand the influence of vegetation encroachment on the growth and sprouting rate. We monitored two plant groups: plants completely shaded by vegetation and plants exposed to the sun, each consisting of 10 individuals. We defined an individual as a cluster since vegetative sprouting generates several branches. To determine whether the plant was shaded or exposed to the sun, a light meter was used at the plant’s maximum height. If the measurement exceeded 2000 lux, the plant was considered exposed to the sun. The width and height measurements of the largest cluster of each individual were recorded, in addition to counting the number of sprouts. Measurements were taken every six months: an initial measurement in October 2022, a new measurement in April 2023 (summer growth interval), and a final measurement in October 2023 (winter growth interval). A digital caliper was used for the measurements. We tested the normality of data using the Shapiro–Wilk test and compared the treatment groups by ANOVA at 5%. We used the software RStudio 2023.03.1 [37] to perform the statistical analyses.

## 5. Conclusions

We hypothesize that *P. rechensis* would be pollinator dependent, and, owing to the gathered evidence, this cactus indeed relies on native insects (especially solitary bees) for pollination services. Notably, and in contrast to preceding literature on the genus [13,14,16], *P. rechensis* is self-incompatible. In practical terms, both features (pollinator dependency and self-incompatibility) have important consequences: (1) any attempt to preserve/manage the remaining specimens should consider, in addition, the preservation of suitable pollinator habitats; and (2) since cross-pollination is mandatory to seed production, attempts should be made to increase the number of individuals in the two previously known *P. rechensis* populations (today collectively reduced to six individuals). This could be achieved by transplanting/translocating either vegetative divisions of plants from other populations or seedlings produced under cultivation. The performance of such transplants could be tracked for a number of years to compare their relative efficacy. Our third hypothesis proposed that germination could be affected by temperature (as already observed in other *Parodia* spp. [13]), and, in fact, the optimal temperature is 20 °C, and a significant germination inhibition was observed at 30 °C. Finally, we hypothesized that vegetative growth could be enhanced by sunlight. This hypothesis was rejected, with plants in shadier places actually growing temporarily higher but not necessarily producing more offshoots or growing more in width. *Parodia rechensis* is an endemic species of Atlantic rainforest from southern Brazil with restricted distribution. The discovery of the new population with more than 450 individuals shed light on the conservation of the species.

The current increase in mean maximum temperatures in Caxias do Sul and the moderate natural low fruiting success suggest a worrying future for the last populations of *P. rechensis*. The management in situ and ex situ of this threatened cactus arises as a logical solution. Other than protecting the remnant populations, we also strongly suggest cultivating the species ex situ, promoting manual cross-pollination to improve the genetic pool. As suggested above, the produced seedlings could be introduced into the natural populations. Even though colony growth in exposed or shaded areas seems indifferent, we suggest introducing the seedlings nearby larger nurse plants that may promote more protection and shadow for the younger plants [38]. In addition, more long-term studies are needed to understand whether this light condition affects flower and fruit production. Finally, an effort to locate potentially overlooked populations is extremely desirable.

## Figures and Tables

**Table 1:** Interactions of pollinators with sexual whorls (stamens and stigma) and duration of interaction.

Species (Order, Family)	Only Stamens	Only Stigma	Both Whorls	Total Interactions	Interaction Duration (s ± sd)
*Augochlora* sp. (Hymenoptera; Halictidae)	10	4	53	67	12.63 (±5.04)
*Ceratina* sp. (Hymenoptera; Apidae)	6	2	17	25	7.91 (±6.34)
Sp. 1 (Coleoptera; Melyridae)	9	0	2	12	371.88 (±49.41)
Sp. 2 (Diptera; Syrphidae)	2	10	0	12	5.11 (±0.71)

**Table 2:** Reproductive system tests in bagged treatments and seeds produced in *Parodia rechensis*. There is no significant difference between natural and manual cross-pollination treatments for fruiting success and seeds produced.

Treatment	Manual Cross-Pollination	Manual Self-Pollination	Natural Pollination	Spontaneous Self-Pollination
Fruiting success	15/35 (42%)	0/31 (0%)	16/30 (53%)	0/32 (0%)
Seeds per fruit (mean ± sd)	31.7 (±15.03)	-	28.9 (±12.31)	-

**Table 3:** Germination test mean values indexes of *Parodia rechensis*. Values with the same letter in the same column do not differ significantly according to the Tukey test at 5%.

Treatment	Germinability (%)	Average Germination Time (Days)	Synchronization Index
20 °C	59 A	12.089 A	3.127 A
25 °C	42 AB	9.202 B	1.911 B
30 °C	25 B	10.590 A	2.293 AB

## Data Availability

Data are available upon request from the correspondent author.

## Supplementary Materials

The following supporting information can be downloaded at: https://www.mdpi.com/article/10.3390/plants13202890/s1, Video S1: Pollinators of *Parodia rechensis*.
